# Supplementary material for: Non-invasive optical control of endogenous Ca2+ channels in awake mice
Source: Nat Commun. 2020 Jan 10;11:210. doi: 10.1038/s41467-019-14005-4 (PMC6954201; doi:10.1038/s41467-019-14005-4)
Supplement: Supplementary file 1 — Supplementary Information [file 41467_2019_14005_MOESM1_ESM.pdf]

Supplementary Information

Non-invasive optical control of endogenous Ca^{2+} channels in awake mice

Kim et al.

Supplementary Figure. 1. Sequence alignment of cryptochrome proteins from diverse species.

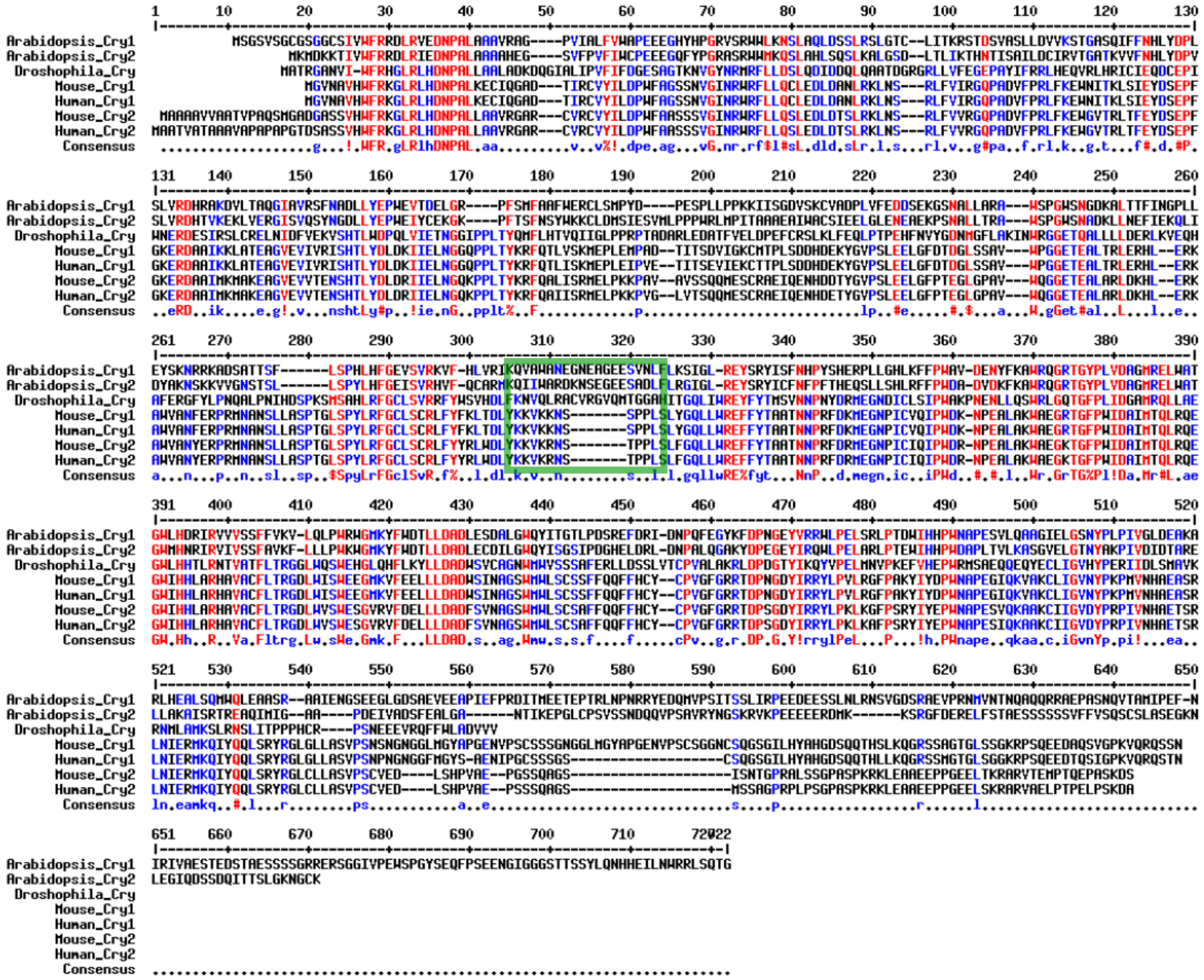

Sequence alignment of cryptochrome proteins from *Arabidopsis thaliana*, *Drosophila melanogaster*, *Mus musculus* and *Homo sapiens*. Sequence conservation from highest to lowest order is colored in red, blue and black. Potential protrusion loop of Arabidopsis cryptochrome for dimerization is marked by a green box.

Supplementary Figure. 2. Tracing self-dissociated CRY2 mutants based on the crystal structures of *Drosophila* and *Arabidopsis* cryptochrome.

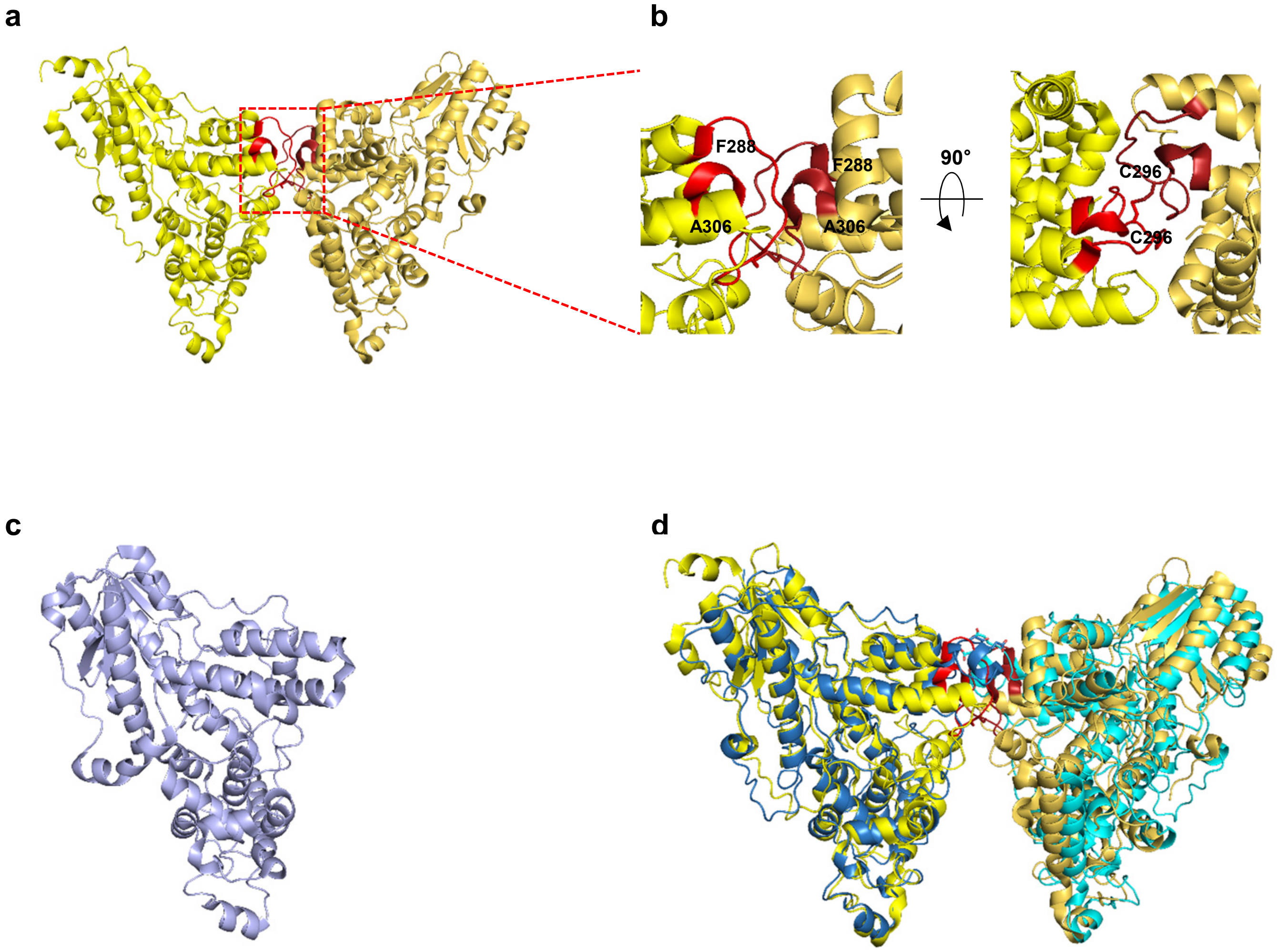

(a) Crystal structure of full-length *d*CRY represented as a dimeric form (PDB Code 4K03). **(b)** Close-up view of the protrusion loop (Phe288–Ala306) at the dimeric interface of *d*CRY shown in red and the 90° rotated view to show the disulfide bridge between Cys296 within the loop. **(c)** Crystal structure of *At*CRY1 (PDB code: 1U3D) shown in light blue, which was used as the model template for *At*CRY2 modeling. **(d)** Overlap of dimeric *d*CRY structure (yellow and orange) with *At*CRY2 model structure (blue and cyan).

Supplementary Figure. 3. Intracellular calcium level in individual cells expressing OptoSTIM1 variants.

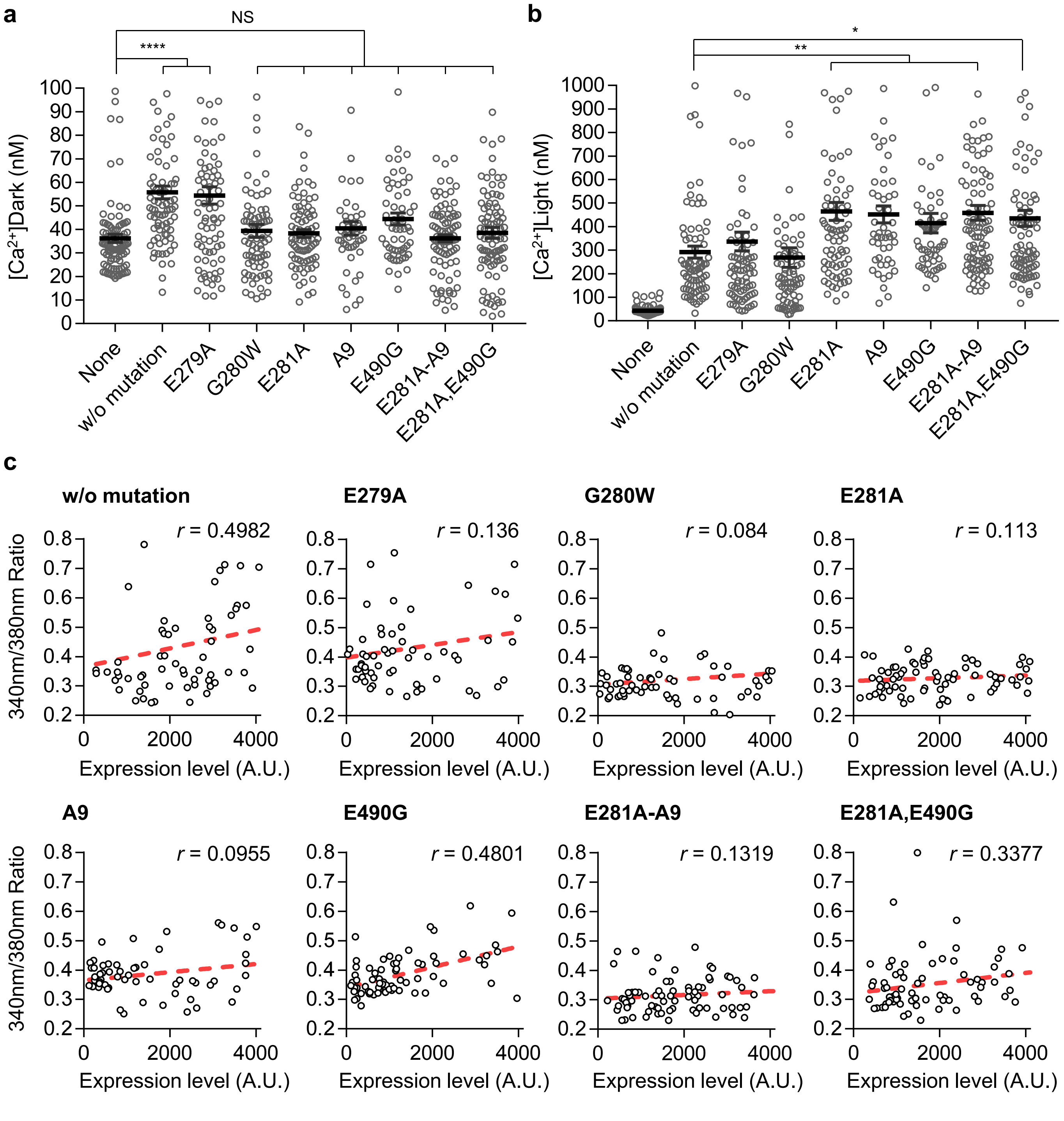

Graphs representing basal **(a)** and light-induced elevated calcium level **(b)** measured by Fura-2. Gray dots indicate individual cells. $n \geq 50$ cells in each group. Error bar, s.e.m. Statistical significance was obtained by Tukey's test (NS = Not significant; $*P < 0.05$; $**P < 0.01$; $****P < 0.0001$). **(c)** Graphs showing emission ratio of Fura-2 dye excited at wavelength of 340 and 380 nm according to the expression level of each variant in HeLa cells. Linear regression lines was presented as red dotted lines. ($n \geq 60$ cells in each graphs, r is spearman correlation)

Supplementary Figure. 4. Activation and deactivation kinetic properties of OptoSTIM1 variants.

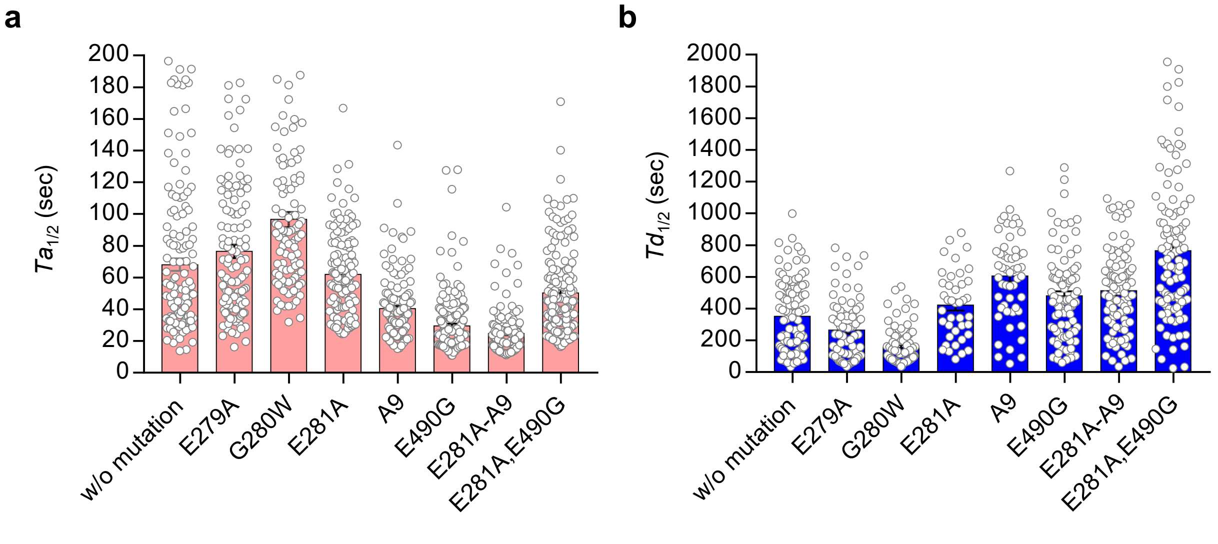

Graphs representing half-maximal time for reaching saturated R-GECO1 intensity upon light stimulation **(a)** and basal R-GECO1 intensity in dark **(b)** for each designated variants. Gray dots indicate individual cells. $n \geq 50$ of each variants. Cells expressing each OptoSTIM1 variants were exposed blue light for 1 min at 5 sec intervals in 500 μW mm^{-2} light density. Error bars, s.e.m.

Supplementary Figure. 5. Measurement of phosphorylated CREB level in cells expressing OptoSTIM1 variants.

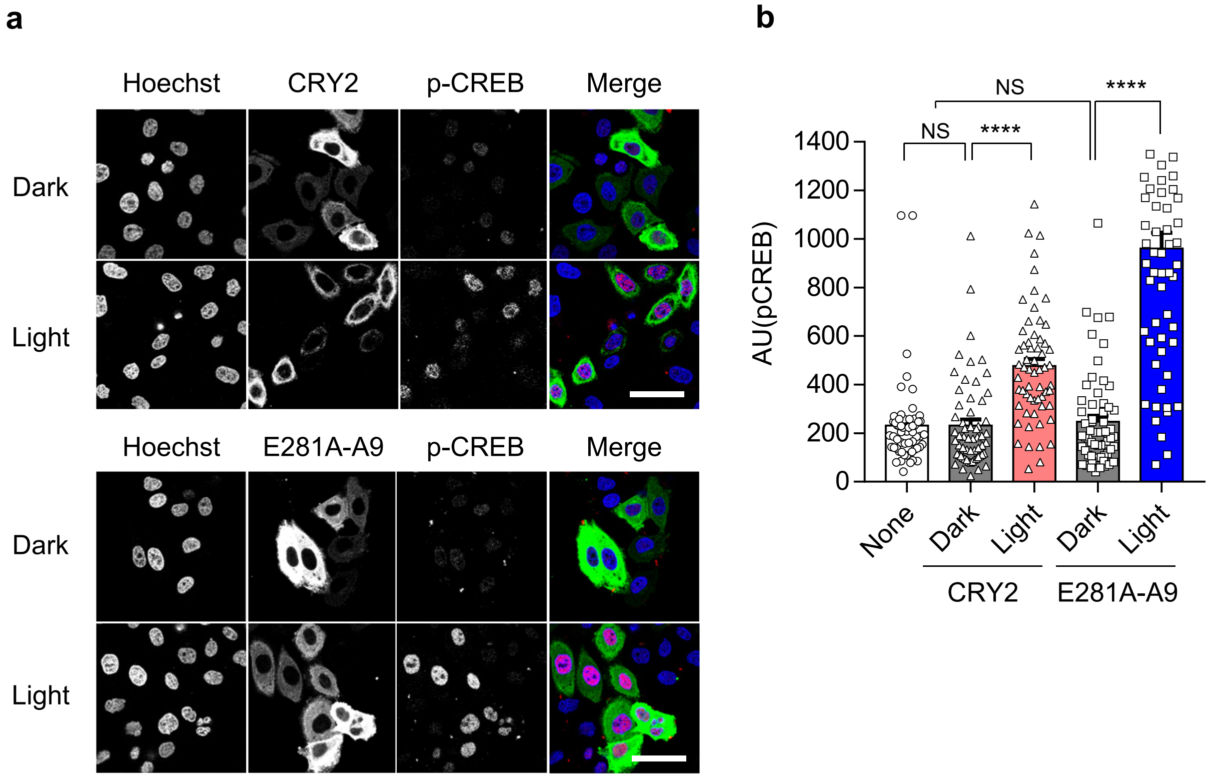

(a) Fluorescence images showing immunostanined p-CREB in HeLa cells either transfected with OptoSTIM1(CRY2) or OptoSTIM1(CRY2^{E281A}-A9) in dark or light stimulated condition. Scale bars, 50 µm. **(b)** Graph indicating average fluorescent intensity of p-CREB shown in a. n = 60 in each group. ****$P < 0.0001$ for Dark versus Light in cells expressing OptoSTIM1 and ****$P < 0.0001$ for Dark versus Light in cells expressing OptoSTIM1(CRY2^{E281A}-A9) by Student's two-tailed *t*-test. NS = not significant. Error bars, s.e.m.

Supplementary Figure. 6. Efficiency of Ca^{2+} influx induced by OptoSTIM1 variants under stimulation with different light densities.

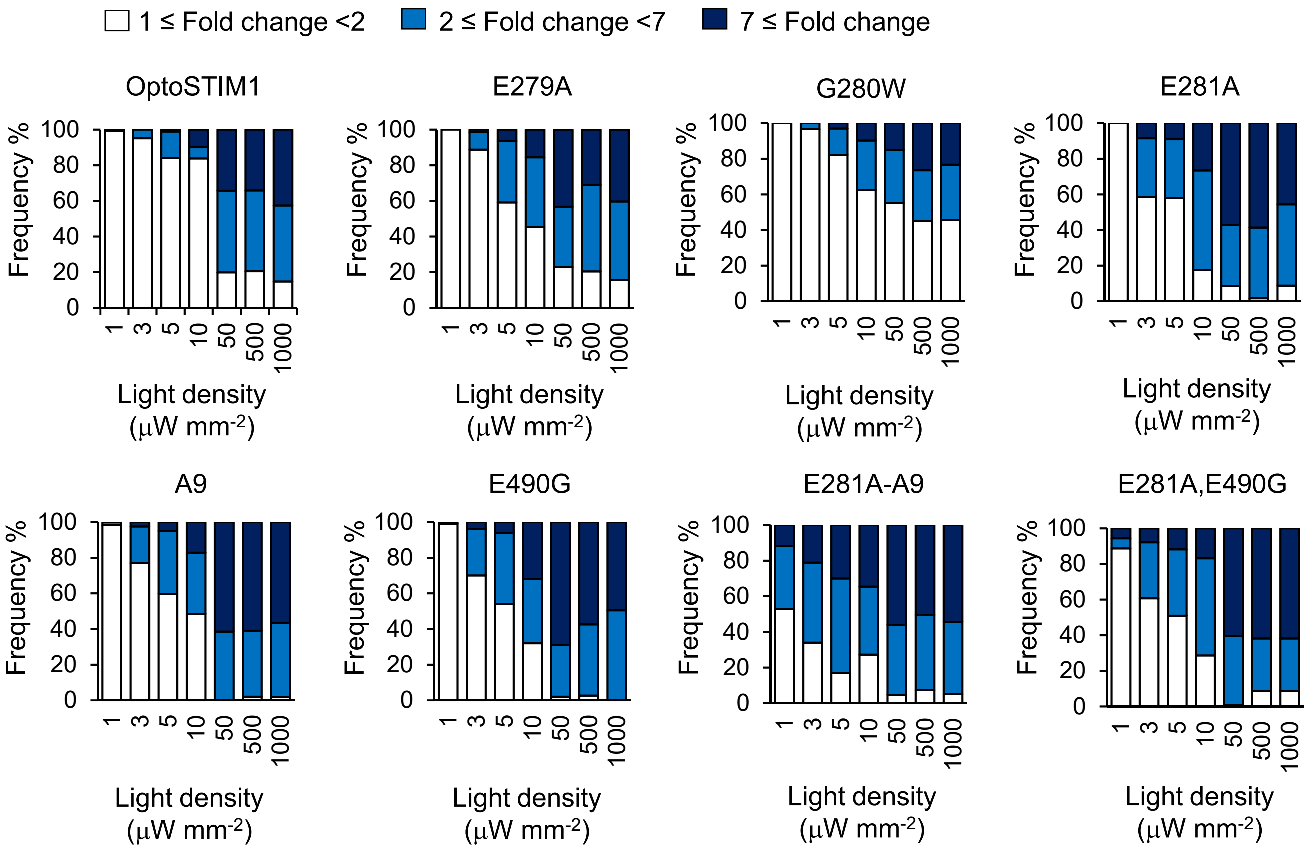

Graphs showing population of cells at indicated ranges of fold changes of R-GECO1 intensity in HeLa cells expressing each OptoSTIM1 variant. Cells were illuminated by blue light for 1min at 5 sec intervals and maximal intensity of R-GECO1 in each cell was normalized by fluorescence intensity (at $t = 0$). Frequency (%) was calculated by dividing the number of cells in each range of maximal fold-change by the number of total cells in each group. ($n \geq 100$ in each bar graphs)

Supplementary Figure. 7. Light dose-dependent maximal Ca^{2+} increase by OptoSTIM1 variants.

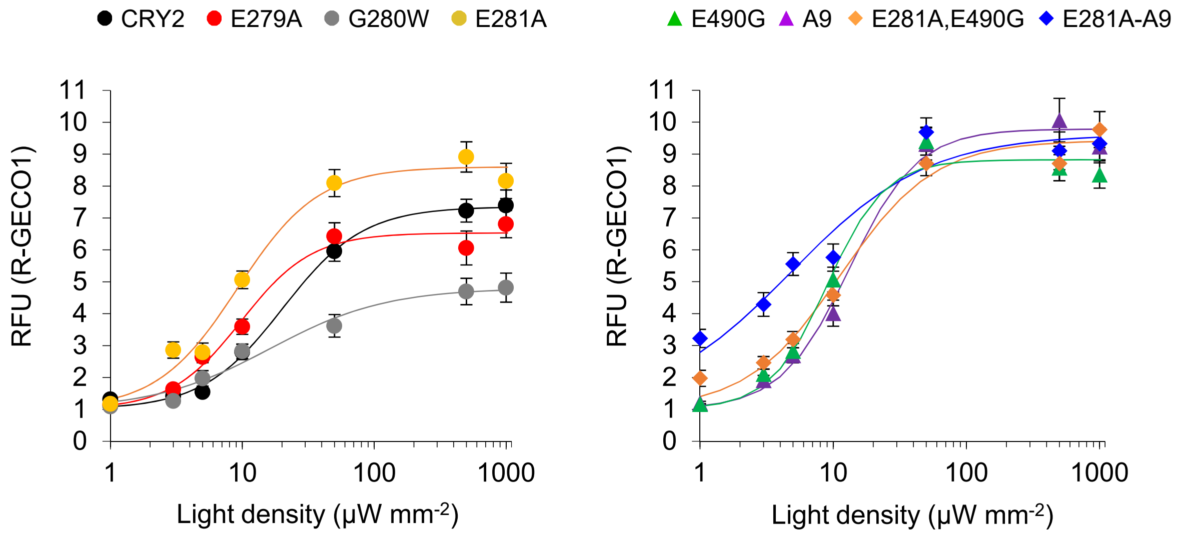

Graph showing averaged maximal R-GECO1 fluorescence level at diverse light-density. Cells were illuminated by blue light for 1 min at 5 sec intervals and maximal intensity of R-GECO1 in each cell was normalized by fluorescence intensity (at $t = 0$). ($n \geq 100$ for each variant). Error bars, s.e.m.

Supplementary Figure. 8. Activation and deactivation kinetics of OptoSTIM1 variants activated under various light densities.

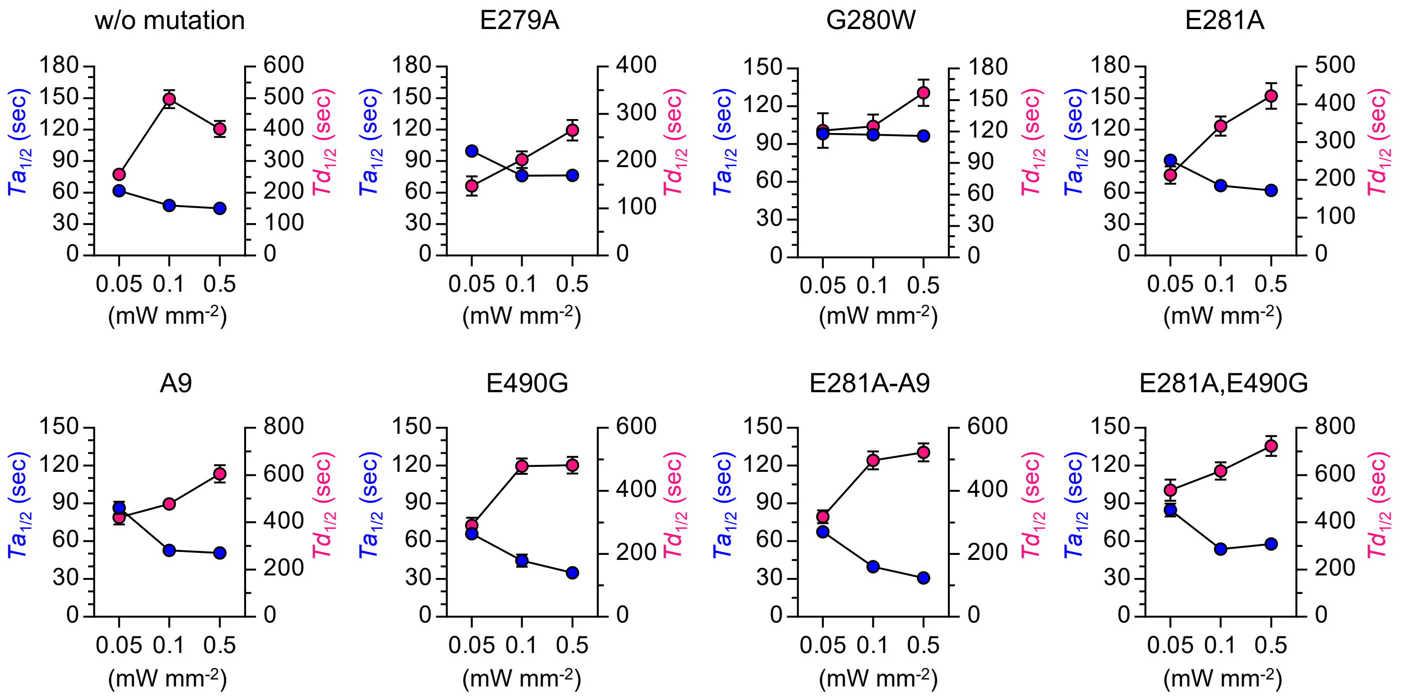

Graphs representing half-maximal time for reaching saturated R-GECO1 intensity upon light stimulation (left *y*-axis) and basal R-GECO1 intensity in dark (right *y*-axis) for each designated variant in disperse condition of light density. $n \geq 40$ for each variant. Cells were exposed to blue light for 1 min at 5 sec intervals at designated light density. Error bars, s.e.m.

Supplementary Figure. 9. Negligible effect of photoactivation of R-GECO1 in assessing Ca^{2+} influx level induced by activated monSTIM1.

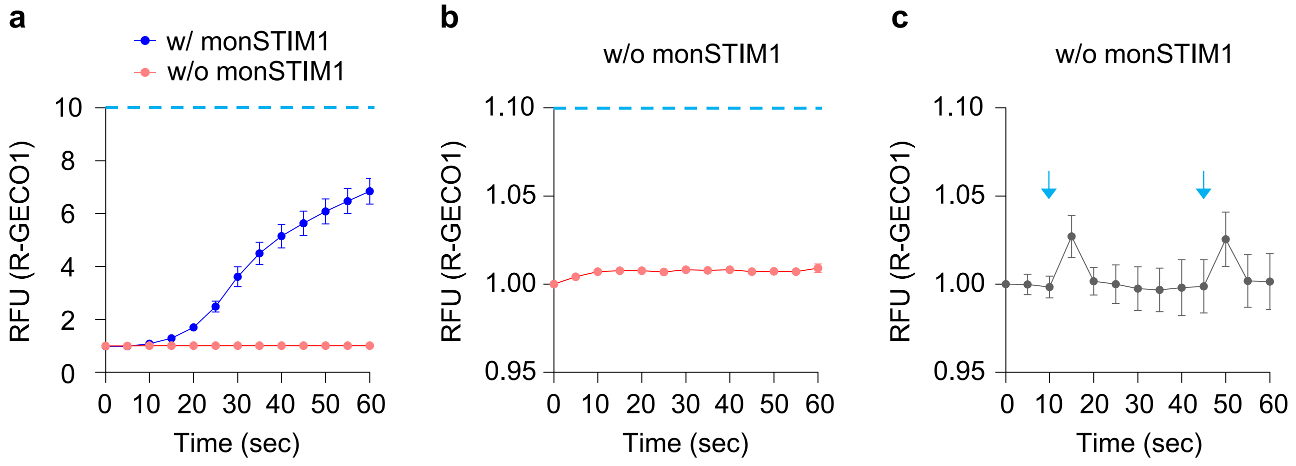

Graphs showing fold change in fluorescence intensity of R-GECO1 based on time-lapse imaging. **(a)** HeLa cells expressing R-GECO1 with or without monSTIM1 was simultaneously imaged with 100 µW mm^{-2} density of 488 nm light with 561 nm light at 5-seconds intervals. Error bars, s.e.m. **(b)** Zoomed-in version of highlighted graph in **a**. **(c)** Photoactivatable property of R-GECO1. Blue light was stimulated at time points indicated by blue arrows (100 µW mm^{-2} for 500 ms). Error bars, s.e.m.

Supplementary Figure. 10. Fundamental properties of monSTIM1

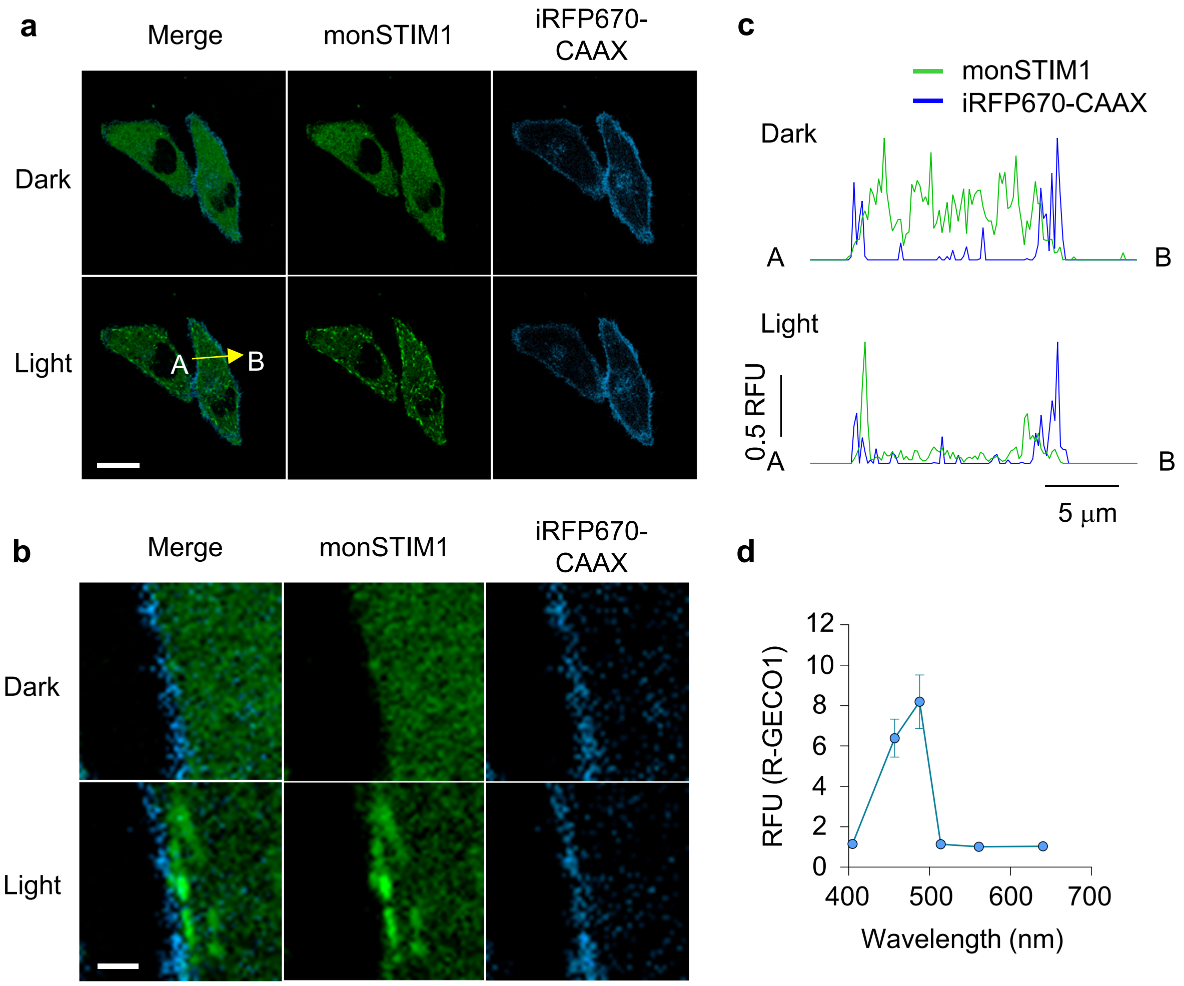

(a) Fluorescence images showing subcellular distribution of monSTIM1 upon blue light irradiation. HeLa cells co-expressing iRFP670-PM(KRas4B tail) were exposed by blue light for 5 min at 5 sec intervals. Scale bar, 20 μm. **(b)** Magnified images showing light dependent translocation of monSTIM1 to the plasma membrane. Scale bar, 2 μm. **(c)** Graphs representing normalized fluorescence intensity of monSTIM1 (green) and iRFP670-PM (blue) corresponding to the yellow line in panel **a**. **(d)** Light spectrum for activating monSTIM1. HeLa cells were exposed to light for 1 min at 5 sec intervals at designated wavelength of light (405, 457, 488, 514, 561 and 640 nm respectively). Error bars, s.e.m.

Supplementary Figure. 11. Analysis of oligomeric properties of STIM1-fused proteins

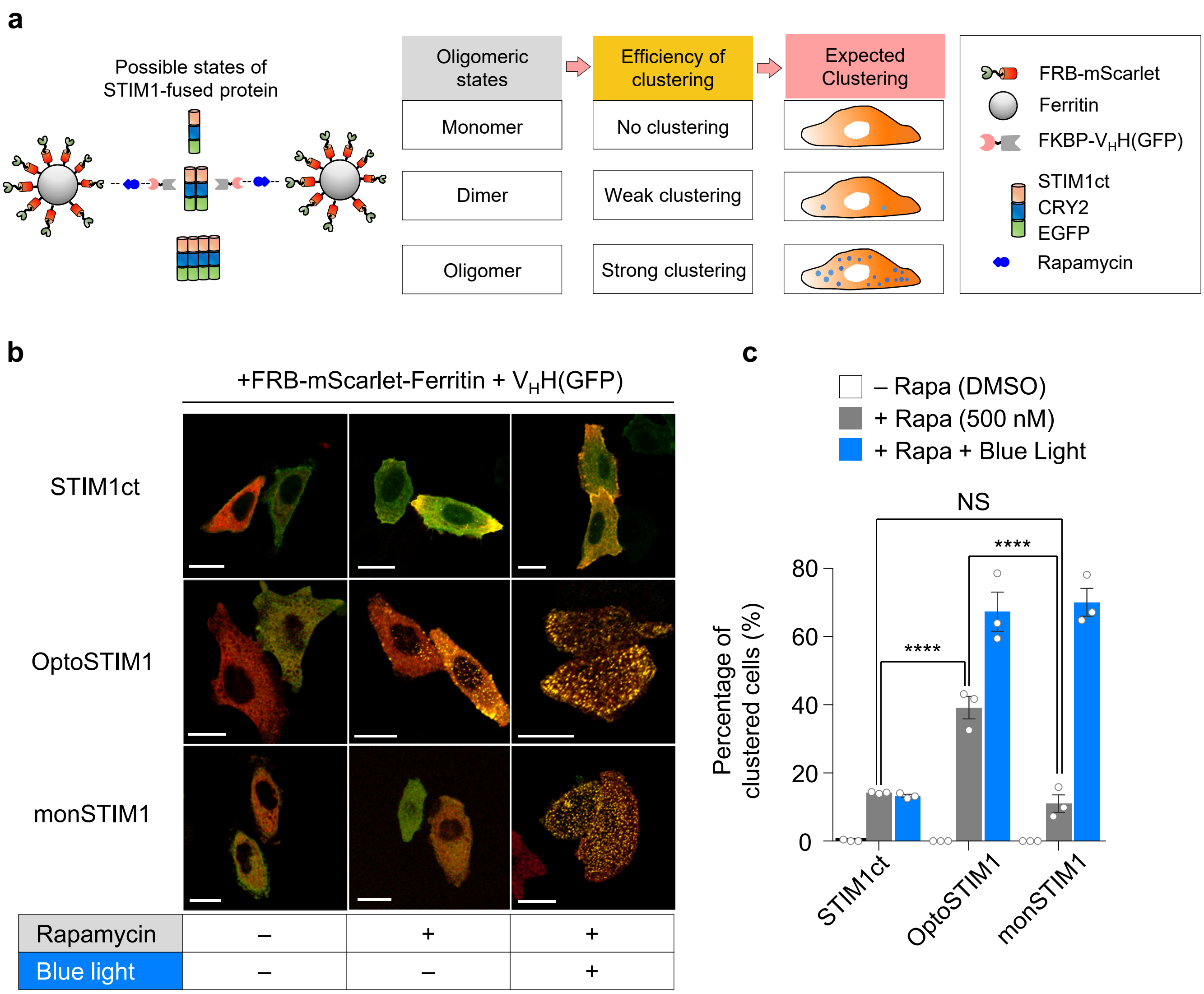

(a) Schematic depiction of InCell SMART-i for assessing interaction properties of CRY2-STIM1ct (a.a 238–685) with or without blue light illumination. **(b)** Fluorescence images showing intracellular cluster formation upon rapamycin treatment (500 nM) in HeLa cells co-expressing FKBP-V_HH(GFP), FRB-mScarlet-Ferritin, and EGFP tagged STIM1ct, OptoSTIM1, or monSTIM1 both in dark and light. Scale bar, 20 µm **(c)** Graph indicating percentage of clustered cells at each described condition. Statistical significant was obtained by Tukey's test, ****$P < 0.0001$; NS = Not significant. Error bars, s.e.m.

Supplementary Figure. 12. Application of light sensitive CRY2 in optical activation of fibroblast growth factor receptor 1.

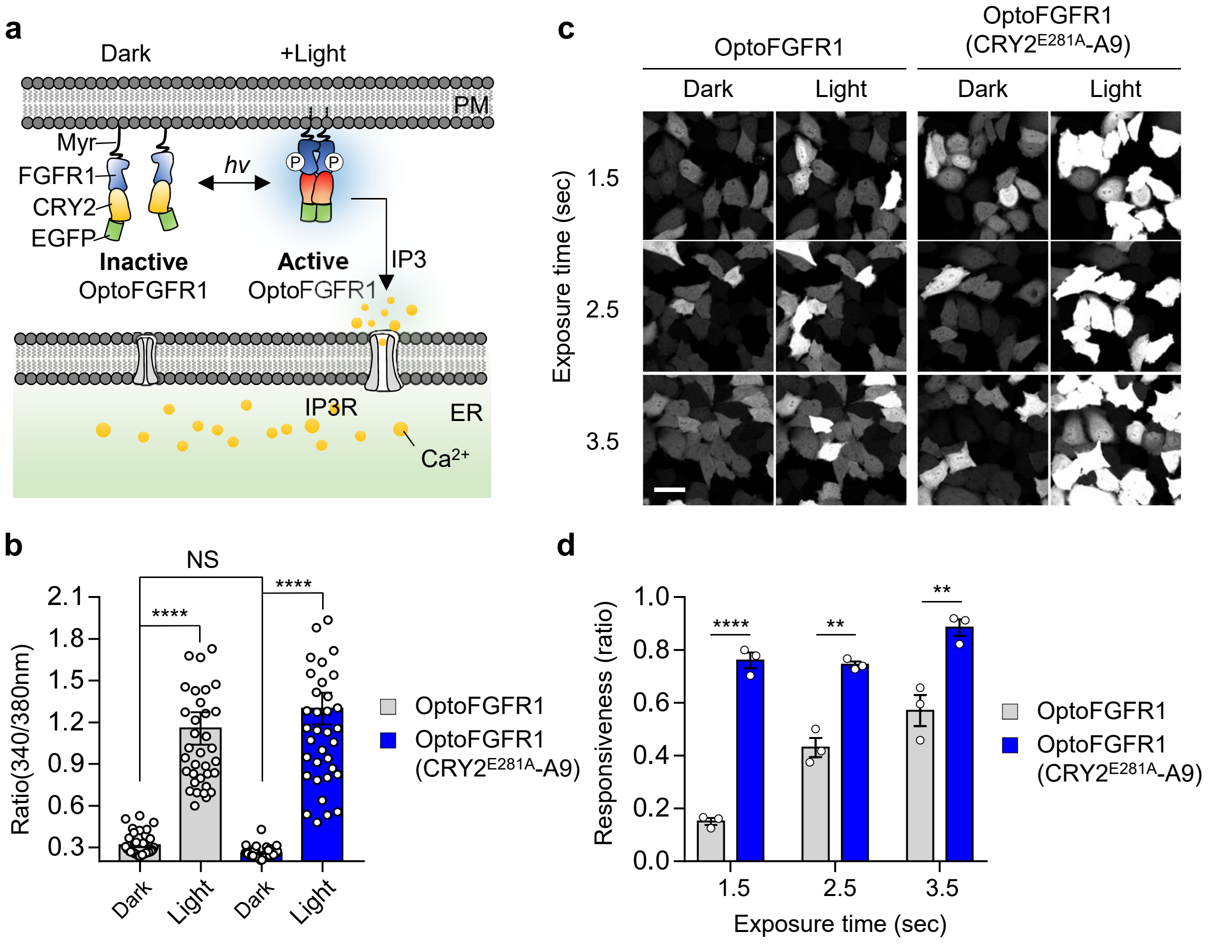

(a) Schematic working mechanism of OptoFGFR1 system. **(b)** Graphs showing emission ratio of Fura-2 dye (340/380 nm) before or after light illumination. Statistical significant was obtained by Tukey's test; NS = Not Significant compared to OptoFGFR1 without light; $^{****}P < 0.0001$. Error bars, s.e.m. **(c)** Representative fluorescence images expressing either OptoFGFR1(CRY2) or OptoFGFR1 ($CRY2^{E281A}$-A9) showing intensity of R-GECO1 upon various exposure time (488nm, 3 μW mm^{-2}, 1.5/2.5/3.5 sec exposure). Scale bar, 20 μm. (d) Graph showing light responded population of cells in each light condition. Responsiveness ratio indicate cells with light-mediated increment of R-GECO1 intensity over total cells. Statistical significant was obtained by Student's two-tailed *t*-test; $^{**}P < 0.01$; $^{****}P < 0.0001$. Error bars, s.e.m.

Supplementary Table. 1. Characteristics of OptoSTIM1 variants.

Mutant type	Basal $[Ca^{2+}]_i$ (nM)	Max $[Ca^{2+}]_i$ (nM)	$Ta_{1/2}$ (sec)	$Td_{1/2}$ (sec)
Original OptoSTIM1	55.8 ± 2.7	292 ± 25.3	68.1 ± 3.9	352.1 ± 22.5
CRY2(E279A)	54.4 ± 3.6	336.7 ± 39.7	76.6 ± 4.2	257.8 ± 29.5
CRY2(G280W)	39.3 ± 2.7	268.7 ± 41.2	96.6 ± 4.7	159.2 ± 17.0
CRY2(E281A)	38.2 ± 1.7	464.6 ± 38	62.0 ± 2.2	422.8 ± 33.8
CRY2(A9)	40.5 ± 2.9	451.8 ± 36.5	40.4 ± 1.6	605.7 ± 37.0
CRY2(E490G)	44.4 ± 2.5	414.8 ± 41.6	29.6 ± 1.3	481.4 ± 26.8
CRY2(E281A-A9)	36.2 ± 1.5	458.3 ± 31.2	24.8 ± 1.0	513.5 ± 25.8
CRY2(E281A,E490G)	38.5 ± 2.3	479.7 ± 45.5	50.4 ± 2.2	764.7 ± 41.3

Basal $[Ca^{2+}]_i$ (nM) represents cytosolic Ca^{2+} concentration without photoactivation ± s.e.m. Max $[Ca^{2+}]_i$ (nM) indicates maximal cytosolic Ca^{2+} concentration upon light illumination ± s.e.m. $Ta_{1/2}$ (sec) and $Td_{1/2}$ (sec) represent half-maximal time for reaching saturated R-GECO1 intensity upon light stimulation and basal R-GECO1 intensity in dark, respectively. Light-power density used was 500 µW mm^{-2}.

Supplementary Figure. 13. The LED array device for illuminating mice in homecage.

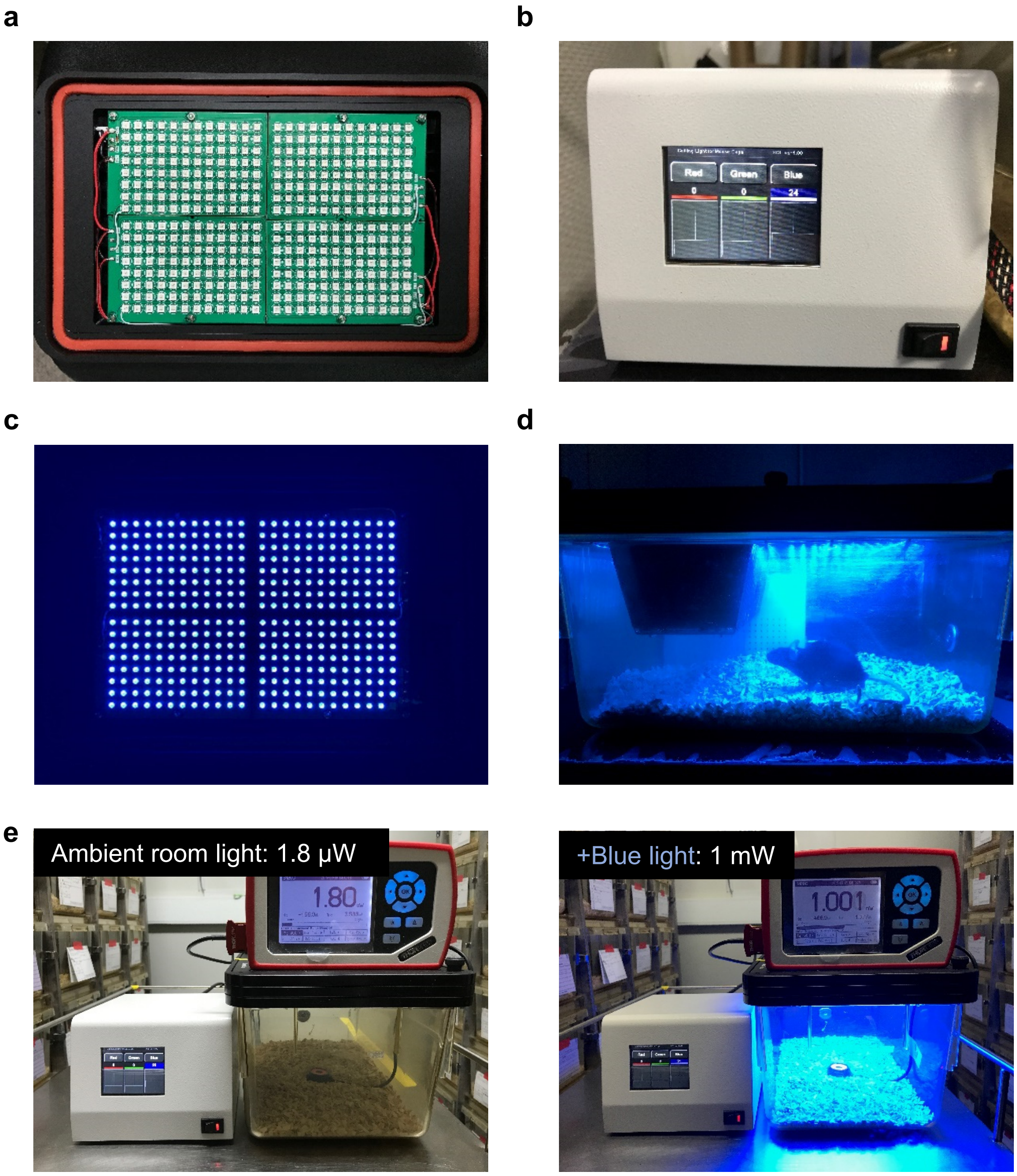

(a) Customized LED arrays attached on cage lid. Total 384 LED bulbs were used for illuminating light. **(b)** Control panel for modulating light density **(c)** Image depicting turned on LED-bulbs in **a**. **(d)** A picture showing side-view of LED cage. **(e)** Representative pictures showing light density of experimental condition of either ambient room light (1.8 μW cm^{-2} density, Left) and light (473 nm at 1 mW cm^{-2} density, Right).

Supplementary Figure. 14. Locomotion and anxiety level of mice expressing OptoSTIM1s in the right ACC.

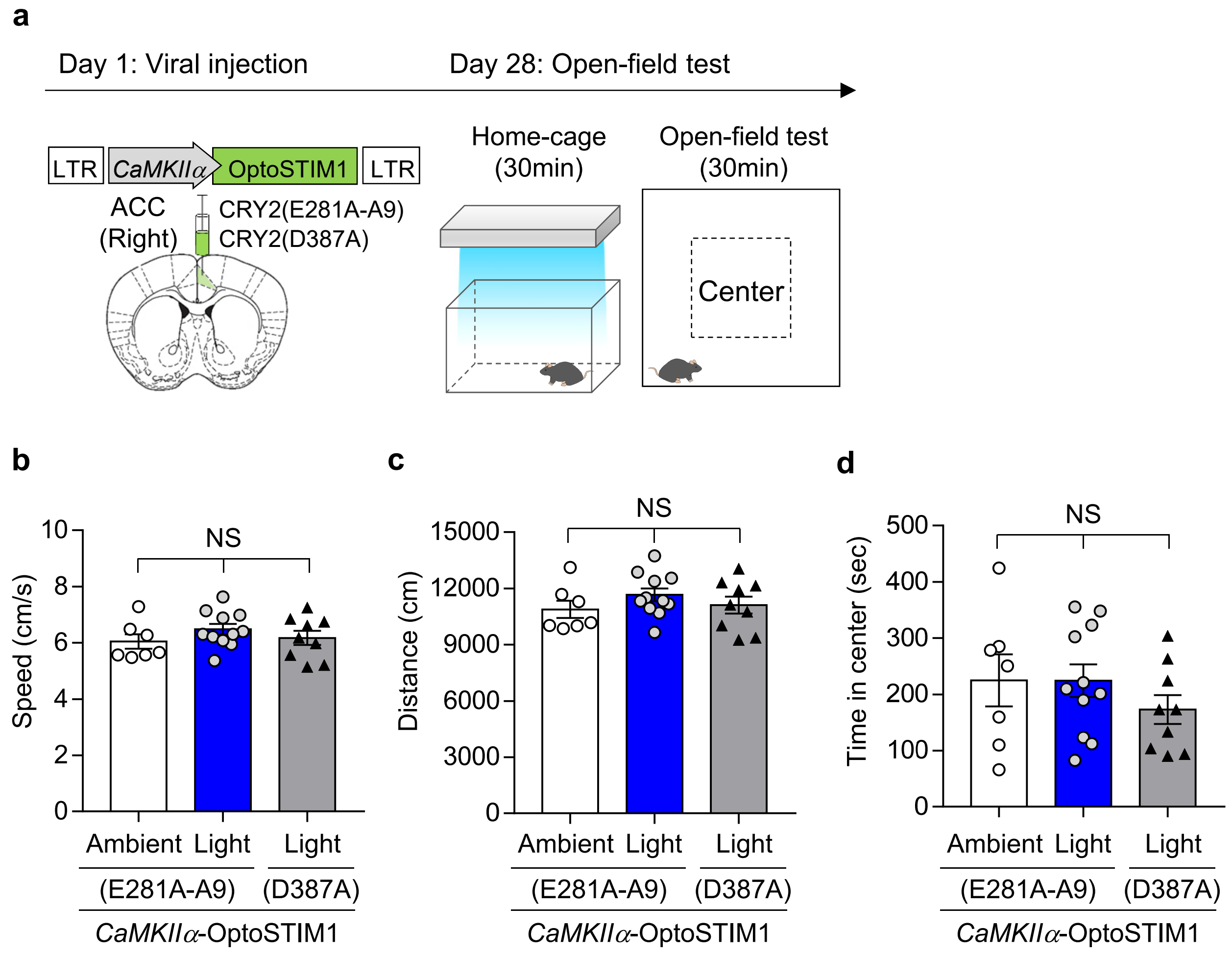

(a) Schematic depiction of open-field test. **(b, c, d)** Graphs indicating general locomotor activity including speed (cm/sec), total distance (cm), and anxiety level measured by time of mice residing in the center during 30 min exploration period. Center area was defined as 20 x 20 cm square in the middle of open-field box. n = 7 (Ambient, E281A-A9); n = 11 (Light, E281A-A9); n = 9 (Light, D387A). Statistical significance was obtained by Tukey's test; NS = Not significant. Error bars, s.e.m.
