# Supplementary material for: Non-invasive optical control of endogenous Ca2+ channels in awake mice
Source: Nat Commun. 2020 Jan 10;11:210. doi: 10.1038/s41467-019-14005-4 (PMC6954201; doi:10.1038/s41467-019-14005-4)
Supplement: Supplementary file 3 — Description of Additional Supplementary Files [file 41467_2019_14005_MOESM3_ESM.pdf]

Description of Additional Supplementary Files

File name: Supplementary Movie 1

Description: Monitoring behavior of a mouse before and after blue-light irradiation in the customized light-illuminating cage. Recorded behavior of a freely moving mouse residing in the homecage with a customized LED lid. Mouse in ambient condition (Left; 0 min to 30 min) was exposed to blue-light at 1 mW cm^{-2} density (Right; 30 min to 60 min).
