# Supplementary material for: Non-invasive optical control of endogenous Ca2+ channels in awake mice
Source: Nat Commun. 2020 Jan 10;11:210. doi: 10.1038/s41467-019-14005-4 (PMC6954201; doi:10.1038/s41467-019-14005-4)
Supplement: Supplementary file 5 — Reporting Summary [file 41467_2019_14005_MOESM5_ESM.pdf]

nature research

Corresponding author(s): Won Do Heo
Last updated by author(s): Nov 14, 2019

Reporting Summary

Nature Research wishes to improve the reproducibility of the work that we publish. This form provides structure for consistency and transparency in reporting. For further information on Nature Research policies, see Authors & Referees and the Editorial Policy Checklist.

Statistics

For all statistical analyses, confirm that the following items are present in the figure legend, table legend, main text, or Methods section.

n/a	Confirmed	
☐	☒	The exact sample size (*n*) for each experimental group/condition, given as a discrete number and unit of measurement
☐	☒	A statement on whether measurements were taken from distinct samples or whether the same sample was measured repeatedly
☐	☒	The statistical test(s) used AND whether they are one- or two-sided<br>*Only common tests should be described solely by name; describe more complex techniques in the Methods section.*
☒	☐	A description of all covariates tested
☒	☐	A description of any assumptions or corrections, such as tests of normality and adjustment for multiple comparisons
☐	☒	A full description of the statistical parameters including central tendency (e.g. means) or other basic estimates (e.g. regression coefficient) AND variation (e.g. standard deviation) or associated estimates of uncertainty (e.g. confidence intervals)
☐	☒	For null hypothesis testing, the test statistic (e.g. *F*, *t*, *r*) with confidence intervals, effect sizes, degrees of freedom and *P* value noted<br>*Give P values as exact values whenever suitable.*
☒	☐	For Bayesian analysis, information on the choice of priors and Markov chain Monte Carlo settings
☒	☐	For hierarchical and complex designs, identification of the appropriate level for tests and full reporting of outcomes
☒	☐	Estimates of effect sizes (e.g. Cohen's *d*, Pearson's *r*), indicating how they were calculated

Our web collection on statistics for biologists contains articles on many of the points above.

Software and code

Policy information about availability of computer code

Data collection	Image data was collected by Nikon imaging software (NIS-elements AR 64-bit v 4.13, Laboratory Imaging) and Freeze frame software.
Data analysis	Imaging data was analyzed in Nikon imaging software 4.13, ImageJ software 1.50b or Matlab R2017a. Scripts were written in Matlab R2017a. Data was analyzed in Graphpad Prism 6 and Microsoft Excel.

For manuscripts utilizing custom algorithms or software that are central to the research but not yet described in published literature, software must be made available to editors/reviewers. We strongly encourage code deposition in a community repository (e.g. GitHub). See the Nature Research guidelines for submitting code & software for further information.

Data

Policy information about availability of data

All manuscripts must include a data availability statement. This statement should provide the following information, where applicable:
- Accession codes, unique identifiers, or web links for publicly available datasets
- A list of figures that have associated raw data
- A description of any restrictions on data availability

The authors declare that the data supporting the findings of this study are available within the paper and its supplementary information files.

Field-specific reporting

Please select the one below that is the best fit for your research. If you are not sure, read the appropriate sections before making your selection.

☒ Life sciences ☐ Behavioural & social sciences ☐ Ecological, evolutionary & environmental sciences

Life sciences study design

All studies must disclose on these points even when the disclosure is negative.

Sample size	Sample sizes were not predetermined. At least 3 biological replicates were performed for most cases, unless noted in the figure legend.
Data exclusions	Related to Fig. 1, if cells showed excessive expression of R-GECO1 with intensity over 1,000 arbitrary unit (NIS-element AR 64-bit, Nikon imaging software), those were excluded in the data analysis.
Replication	All attempts at replications were successful. All results are reported as average of multiple experiments with their variance.
Randomization	The samples were randomly grouped.
Blinding	The investigators were blinded to group allocation during data collection and analysis.

Reporting for specific materials, systems and methods

We require information from authors about some types of materials, experimental systems and methods used in many studies. Here, indicate whether each material, system or method listed is relevant to your study. If you are not sure if a list item applies to your research, read the appropriate section before selecting a response.

Materials & experimental systems

n/a	Involved in the study	
☐	☒	Antibodies
☐	☒	Eukaryotic cell lines
☒	☐	Palaeontology
☐	☒	Animals and other organisms
☒	☐	Human research participants
☒	☐	Clinical data

Methods

n/a	Involved in the study	
☒	☐	ChIP-seq
☒	☐	Flow cytometry
☒	☐	MRI-based neuroimaging

Antibodies

Antibodies used	Anti-Phospho-CREB (Ser133) Rabbit mAb (#9198, CST), Anti-GFP chicken (A10262 ,Thermo Fisher Scientific), Anti-c-Fos rabbit (Ab190289, Abcam), Alexa 594-conjugated anti-rabbit secondary antibody (A-11037, Thermo Fisher Scientific), Alexa 488-conjugated anti-chicken secondary antibody (A-11039, Thermo Fisher Scientific)
Validation	Antibodies were only chosen if there were validated references available. Anti-GFP antibody was validated by detecting exogenously expressed GFP-tagged protein. Anti-c-Fos antibody was validated through behavioral test and detection at the lentivirus delivered sites in sliced mouse brain. Anti-phosphorylated CREB antibody was validated by experiment where HeLa cells were treated with ionomycin to increase intracellular Ca2+ concentration and pCREB level.

Eukaryotic cell lines

Policy information about cell lines

Cell line source(s)	Hela and HEK293 cell lines were acquired from ATCC.
Authentication	None of the cell lines used were authenticated.
Mycoplasma contamination	The cell line was confirmed negative for mycoplasma contamination by PCR-based mycoplasma detection methods.
Commonly misidentified lines (See ICLAC register)	No commonly misidentified cell lines were used.

Animals and other organisms

Policy information about studies involving animals; ARRIVE guidelines recommended for reporting animal research

Laboratory animals	C57BL/6J, Male, 8-13 week-old.
Wild animals	N/A

Field-collected samples	N/A
Ethics oversight	All mice were handled and cared according to the directives of the Animal Care and Use Committee of KAIST (Daejeon, Korea).

Note that full information on the approval of the study protocol must also be provided in the manuscript.
